# Laser treatment for urinary incontinence in elite female athletes analyzed using a discrete mathematics approach

**DOI:** 10.1038/s41598-025-00363-1

**Published:** 2025-05-02

**Authors:** Nobuo Okui

**Affiliations:** 1 Urogynecology, Yokosuka Urogynecology and Urology Clinic, Ootaki 2-6, Yokosuka, 238-0008 Kanagawa Japan; 2 Mathematics, Kanagawa Dental University, Inaoka-cyou 82, Yokosuka, 238-0008 Kanagawa Japan; 3 Urology, Graduate School of Medicine, Juntendo University, Tokyo, Japan

**Keywords:** Stress urinary incontinence, Elite female athletes, Non-ablative Er:YAG laser therapy, Pelvic floor muscle training, Discrete mathematics analytical approach, Network graphs, Personalized medicine, Treatment outcomes, Return to sports, Discrete mathematics, Medical research, Urinary incontinence

## Abstract

Efficient treatment strategies for stress urinary incontinence (SUI) in elite female athletes (EFAs) are crucial for their timely return to sports. This study evaluates the effectiveness and potential drawbacks of non-ablative Er: YAG laser therapy combined with pelvic floor muscle training (PFMT) in treating SUI among EFAs. We employ a discrete mathematics analytical approach using network graphs to identify key factors influencing treatment outcomes and to address the challenges of small sample sizes and unknown variables in this population. Our results demonstrate significant improvements in urinary incontinence symptoms and increased return rates to elite sports activities in the laser treatment group compared to the PFMT-only group. The discrete mathematics approach effectively visualizes the complex relationships between variables and supports the development of personalized treatment plans. This study highlights the potential of laser therapy as an effective treatment option for SUI in EFAs while emphasizing the importance of tailored treatment strategies.

## Introduction

Observational data reflect real-world clinical practice and are valuable for studying rare diseases or limited populations^1–3^. However, analyzing observational data presents challenges due to potential biases and unknown variables. Propensity score (PS) matching addresses these issues, but results vary depending on variables used^3,4^. Multivariate analysis and multiple regression require known variables^5,6^, which is difficult in real-world settings^7,8^. These methodological limitations highlight the need for more advanced, flexible, and interpretable approaches to extract meaningful insights from observational data, particularly in complex or small-scale clinical populations^9,10^.

Advanced causal inference techniques, such as instrumental variable (IV) and difference-in-differences (DiD), address the limitations of traditional methods but have challenges, including complexity, computational burden, and difficulty in interpreting results^11,12^. Network graphs based on discrete mathematics visually capture relationships between variables^13–17^. Especially in small or complex populations, such as elite female athletes (EFAs), this approach allows researchers and clinicians to map out multi-dimensional dependencies and identify key predictors in a way that is both data-driven and clinically applicable^18,19^.

Stress urinary incontinence (SUI) is highly prevalent among EFAs, especially in high-impact sports, with reported rates reaching up to 80%^20^. This condition can impair performance and delay return to competition^21,22^. While pelvic floor muscle training (PFMT) remains the recommended conservative treatment^23,24^, energy-based therapies such as Er: YAG and CO₂ lasers have demonstrated comparable effectiveness in recent studies^21,25,26^. Notably, MRI-based assessments have shown that non-ablative Er: YAG laser therapy can alter the morphology of the urethra from elliptical to circular and increase the thickness of the anterior and posterior vaginal walls, thereby enhancing structural support of the urethra^25^. These mechanical effects may contribute to improved continence while minimizing invasiveness and recovery time, making laser therapy particularly advantageous for EFAs.

The discrete mathematical approach, based on graph theory and network analysis, represents complex relationships between variables as a network graph. In this framework, each node corresponds to a clinical variable (e.g., hormone levels, PFMT frequency), and edges indicate statistical associations or partial correlations. Node size is determined by the variance inflation factor (VIF), reflecting each variable’s relative influence. By analyzing the graph structure—such as shortest paths or node centrality—clinicians can identify key predictors and treatment pathways. This approach enables visual and interpretable decision support, especially in small or complex populations like EFAs^13–17^.

This study aimed to elucidate the effects and risks of laser therapy in treating SUI in EFAs by applying a discrete mathematical framework. Network graphs were used to visualize interactions between clinical variables and to identify key predictors of treatment success. This approach supports personalized medicine by helping determine which patients may benefit most—or least—from laser therapy. Findings may offer new insights into the advantages and limitations of laser-based treatment in EFAs and demonstrate the utility of graph theory in analyzing complex clinical data.

## Results

### Limitations of propensity score matching in evaluating the effectiveness of laser treatment

In our sports clinic outpatient department, we admitted 354 female runners who were active in the elite division of the athletics federation and had experienced severe SUI from 2012 to 2022. All patients were unable to continue their athletic activities because of SUI and provided informed consent for treatment. Three patients had neurogenic bladders. All the patients received PFMT instructions. Fourteen people desired Mid-Urethral Sling (MUS) 9 but claiming that artificial objects inside the body would affect their running form, only 8 underwent the procedure.

**Fig. 1:**
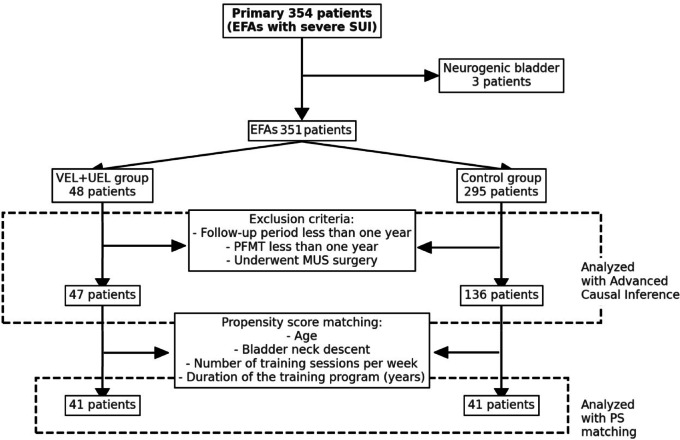
Flow diagram of patient selection and propensity score matching.

The vaginal and urethral non-ablative Er: YAG Laser Therapy (VEL + UEL) group comprised 48 individuals who received laser treatment in addition to PFMT. All but one successfully completed the one-year follow-up period. Patients in the VEL + UEL group did not desire additional MUS surgery after completing VEL + UEL treatment. The control group, which initially received only PFMT, consisted of 295 patients. However, due to four patients requesting MUS and some dropouts, the final count was 136 cases. After conducting PS matching based on age, bladder neck descent (BND), duration of the training program, and number of training sessions per week, 41 individuals were selected for both the VEL + UEL and control groups (Fig. 1). Although PS matching was applied to reduce confounding, the final matched sample size of 41 patients per group may limit the statistical power to detect modest treatment effects. These results should therefore be interpreted with caution. As the findings were derived from a highly specific population of EFAs with severe SUI, generalizability to broader or non-athletic populations may be limited.

Table 1 displays the comparison results after PS matching between the VEL + UEL group and the Control group. The VEL + UEL group consisted of 41 individuals, while the Control group had 41. The comparison was focused on patient background factors such as age and BMI, with statistical significance denoted by p-values. In this comparison, no significant differences were found between the two groups for factors such as age (*p* = 0.9900) and BMI (*p* = 0.878). Similarly, there were no significant differences in other factors such as smoking, alcohol consumption, hypertension, hyperlipidemia, and diabetes between the groups (*p* > 0.05). For anatomical structures implicated in the occurrence of SUI, such as BND (cm) (*p* = 0.550), contractility of levator hiatus (*p* = 0.016), and distensibility of levator hiatus (*p* = 0.742), there were no significant differences noted, suggesting no group differences in these anatomical features. The International Consultation on Incontinence Questionnaire – Short Form (ICIQ-SF), which evaluates personal QOL concerning SUI, showed no significant difference (*p* = 0.102). The measure for excessive training volume in athletes, Relative Energy Deficiency in Sport (RED-S), also showed no significant difference (*p* = 1.00). During the one-year observation period, a decreasing trend was observed in the frequency of PFMT sessions and the weekly training duration, so the mean values were calculated. In the comparative analysis between the two groups, there were no statistically significant differences observed in the frequency of PFMT sessions or the weekly training duration, with p-values of 0.569 and 0.341, respectively.

**Table 1:** Comparison of patient characteristics and clinical variables between the treatment and control groups after propensity score matching.

Variable	Treatment Group (Mean ± SD)	Control Group (Mean ± SD)	*P* value
Age (years)	35.66 ± 6.32	36.45 ± 5.21	0.582
Urinary incontinence since before childbirth	0.10 ± 0.30	0.10 ± 0.31	0.937
Number of births	2.00 ± 0.63	2.03 ± 0.50	0.808
Vaginal delivery	1.00 ± 0.00	1.00 ± 0.00	nan
Cesarean section	0.00 ± 0.00	0.00 ± 0.00	nan
Weekly training days	6.22 ± 1.06	6.34 ± 0.97	0.616
Duration of training (years)	25.20 ± 5.49	25.17 ± 5.04	0.986
Time since last race (years)	0.42 ± 0.16	0.47 ± 0.14	0.156
Running	0.93 ± 0.26	1.00 ± 0.00	nan
Interval Training	0.61 ± 0.49	0.59 ± 0.50	0.846
Core Training	0.56 ± 0.50	0.79 ± 0.41	0.045
Weight Training	0.95 ± 0.22	0.86 ± 0.35	0.195
Headache	0.29 ± 0.46	0.48 ± 0.51	0.108
Back Pain	0.10 ± 0.30	0.03 ± 0.19	0.320
Plantar Fasciitis	0.07 ± 0.26	0.17 ± 0.38	0.204
History of heart disease/arrhythmias, angina	0.02 ± 0.16	0.00 ± 0.00	nan
Asthma or chronic bronchitis	0.02 ± 0.16	0.00 ± 0.00	nan
Fractures or joint injuries	0.05 ± 0.22	0.00 ± 0.00	nan
History of pelvic surgery	0.00 ± 0.00	0.00 ± 0.00	nan
Diabetes	0.00 ± 0.00	0.00 ± 0.00	nan
Thyroid dysfunction	0.00 ± 0.00	0.00 ± 0.00	nan
Menstrual irregularities or amenorrhea	0.07 ± 0.26	0.03 ± 0.19	0.499
RED-S: Relative Energy Deficiency in Sport	0.07 ± 0.26	0.03 ± 0.19	0.499
Depression	0.00 ± 0.00	0.00 ± 0.00	nan
Uterine diseases/endometriosis	0.24 ± 0.43	0.03 ± 0.19	0.017
Ovarian tumor diseases	0.02 ± 0.16	0.00 ± 0.00	nan
Breast cancer	0.00 ± 0.00	0.00 ± 0.00	nan
Neurogenic bladder	0.00 ± 0.00	0.00 ± 0.00	nan
Congenital urethral malformation	0.00 ± 0.00	0.00 ± 0.00	nan
Malformation of the ureteral orifice	0.00 ± 0.00	0.00 ± 0.00	nan
Bladder neck descent (BND) cm	1.87 ± 0.12	1.78 ± 0.09	0.001
Distensibility of levator hiatus (mm)	17.17 ± 0.68	17.24 ± 0.56	0.646
Contractility of levator hiatus (mm)	12.82 ± 0.51	12.47 ± 0.55	0.008
Testosterone (resting, early morning)	54.46 ± 5.88	29.86 ± 7.11	0.000
Estradiol	111.12 ± 104.31	190.07 ± 100.95	0.002
T-cho	110.95 ± 78.95	159.24 ± 45.92	0.004
Hb	13.28 ± 0.79	12.84 ± 0.42	0.008
Pelvic floor muscle exercise duration per day	0.99 ± 0.31	1.03 ± 0.44	0.603
Pelvic floor muscle exercise frequency per week	6.78 ± 0.42	6.10 ± 0.41	0.000
ICIQ-SF	13.61 ± 0.95	12.59 ± 2.35	0.014
1HrPadTest	76.61 ± 14.65	67.38 ± 22.88	0.043

Data are presented as mean ± standard deviation. The treatment group received a combination of pelvic floor muscle training (PFMT) and vaginal/urethral erbium-doped yttrium aluminum garnet (Er: YAG) laser therapy (VEL + UEL), while the control group received PFMT only. P values were calculated using appropriate statistical tests to compare the differences between the two groups. nan indicates that the p value could not be calculated due to the absence of variability in the data.

This study evaluated the effects of combined treatment (PFMT and VEL + UEL) versus PFMT alone on urinary incontinence and athletic performance over one year. The results indicated significant improvements in the treatment group compared with the control group.

**Fig. 2:**
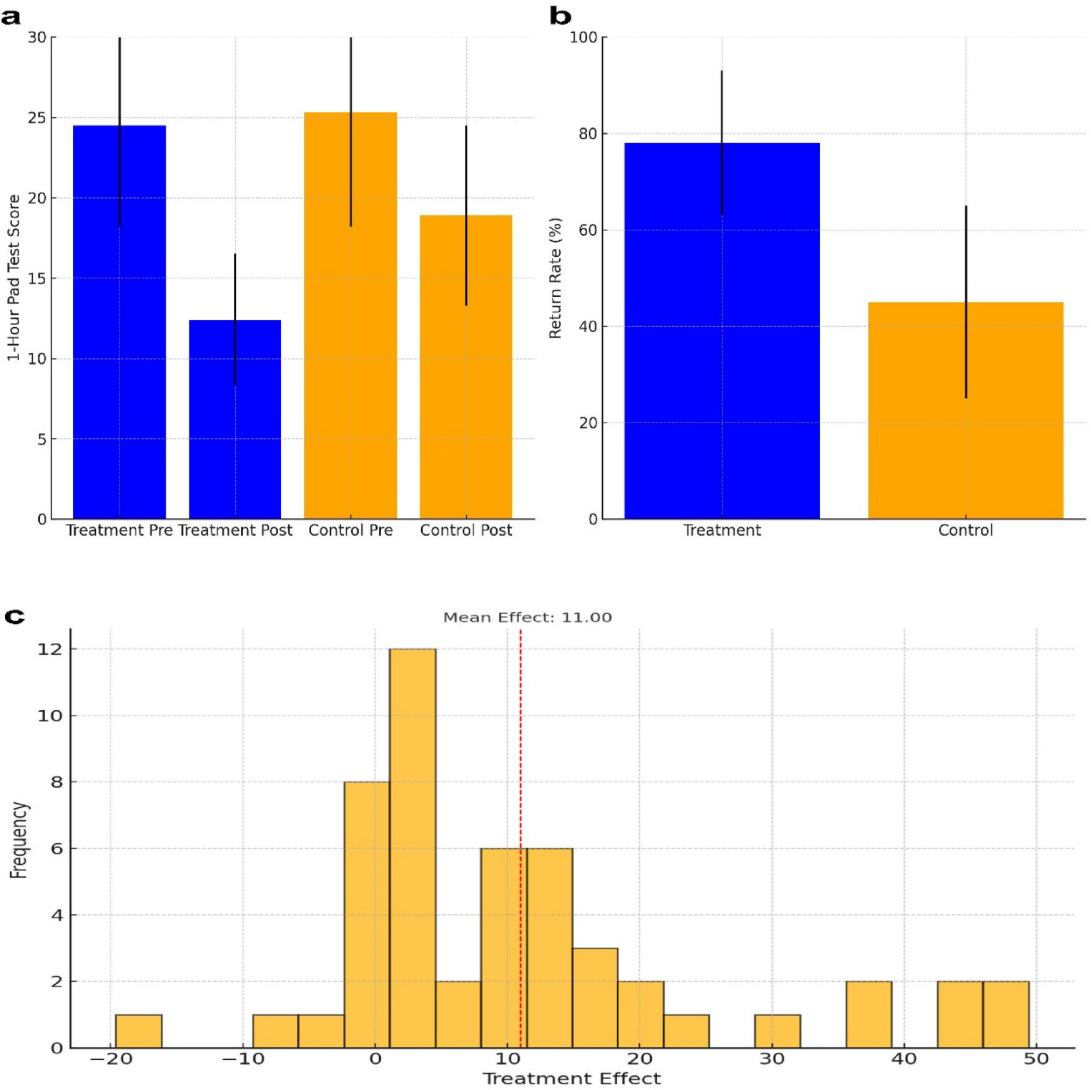
Limitations of Propensity Score Matching in Evaluating the Effectiveness of Laser Treatment. (**a**) Comparison of 1-hour pad test (1HrPadTest) before and after treatment. The vertical axis represents the 1HrPadTest (g), and the horizontal axis shows the treatment groups (Treatment and Control) at pre and post-treatment time points. (**b**) Return to elite sports one year after treatment. The vertical axis represents the return rate (%), and the horizontal axis shows the treatment groups (Treatment and Control). (**c**) The distribution of treatment effects from different covariate sets in the sensitivity analysis. The horizontal axis represents the treatment effect, and the vertical axis shows the frequency of each treatment effect. The red dashed line indicates the mean treatment effect (Mean Effect: 11.00).

For the 1-hour pad test (1HrPadTest), as shown in Fig. 2a, the treatment group showed a marked reduction in scores, with pre-treatment values averaging (24.5 ± 6.3 g) and post-treatment values decreasing to (12.4 ± 4.1 g). This reduction was statistically significant (*p* < 0.0001), demonstrating the effectiveness of the combined treatment in alleviating urinary incontinence symptoms. Similarly, the control group, which received PFMT alone, exhibited a reduction in 1HrPadTest from (25.3 ± 7.1) pre-treatment to (18.9 ± 5.6) post-treatment, with the improvement also being significant (*p* < 0.0001).

In terms of athletic performance, the return rates to elite competitions after one year, as illustrated in Fig. 2b, were significantly higher in the treatment group. The treatment group achieved an average return rate of (78 ± 15%), whereas the control group had a markedly lower return rate of (45 ± 20%). This difference was statistically significant (*p* < 0.0001), underscoring the efficacy of the combined treatment in facilitating the return to high-level athletic activities.

Additionally, the improvement in ICIQ-SF scores over one year further highlights the benefits of combined treatment. The treatment group demonstrated substantial improvement, with scores averaging (12.44 ± 3.70), compared to the control group, which showed an average improvement of only (1.66 ± 3.52). This difference was highly significant (*p* < 0.0001), indicating a significant enhancement in urinary function and overall quality of life in patients in the treatment group.

The combined treatment of PFMT and VEL + UEL significantly improved urinary incontinence symptoms and enhanced the return to elite sports compared to PFMT alone. These findings support the adoption of combined treatment for athletes with urinary incontinence.

The improvement in ICIQ-SF scores was significantly greater in the treatment group (mean: 12.61, 95% CI: 11.55–13.67) compared to the control group (mean: 3.68, 95% CI: 2.55–4.81). Similarly, the 1HrPadTest improvement was markedly higher in the treatment group (mean reduction: 71.37 g, 95% CI: 64.77–77.96) than in the control group (32.78 g, 95% CI: 27.13–38.43), indicating a clinically meaningful benefit from the combined intervention.

#### Sensitivity analysis results

Sensitivity analysis was conducted to assess the robustness of the treatment effect estimates obtained from PS matching. The analysis involved randomly selecting 50 different sets of covariates, each containing 4 variables, to calculate the treatment effects. The results indicated an average treatment effect of 10.9971 with a standard deviation of 14.8087.

The mean treatment effect of approximately 11.0 suggests that the treatment group showed an average improvement of approximately 11 units compared to the control group. However, the large standard deviation of approximately 14.8 indicates significant variability in the treatment effects, implying that the choice of covariates substantially influences the results. This high variability suggests that PS matching results are sensitive to the selection of covariates, highlighting the potential for residual confounding. Figure 2c shows the distribution of the treatment effects across the different covariate sets, illustrating variability in the estimates.

Given these findings, it is crucial to interpret the results with caution, as sensitivity to covariate selection may affect the robustness of the conclusions. While these results suggest the potential effectiveness of VEL+UEL treatment, the sensitivity analysis indicates that unobserved confounders may influence the results. To address this issue and obtain reliable estimates of the treatment effects, further investigations using more sophisticated methods, such as multivariate analysis, are necessary.

### Method for selecting important variables other than laser treatment

#### Pearson’s correlation coefficients

The Pearson’s correlation coefficients between baseline characteristics and the outcomes are summarized in Table 2.

**Table 2:** Pearson’s correlation coefficients between the baseline characteristics and the outcomes at one year and three months.

Variable	Values	Return to sport after 1 year	*P* value (Return to sport)	1HrPadtest (1 year later)	*P* value (Pad test)
Age	37.40 ± 4.72	-0.197 (-2.549–2.156)	0.010	-0.032 (-2.384–2.320)	0.677
Urinary incontinence since before childbirth	0.08 ± 0.27	-0.012 (-0.145–0.120)	0.875	-0.018 (-0.151–0.114)	0.813
Number of births	1.94 ± 0.49	0.015 (-0.227–0.258)	0.841	0.050 (-0.192–0.293)	0.512
Vaginal delivery	1.00 ± 0.00	nan (nan - nan)	nan	nan (nan - nan)	nan
Cesarean section	0.00 ± 0.00	nan (nan - nan)	nan	nan (nan - nan)	nan
Weekly training days	6.16 ± 1.02	0.059 (-0.447–0.566)	0.440	0.039 (-0.467–0.545)	0.612
Duration of training (years)	25.95 ± 4.62	-0.090 (-2.396–2.215)	0.241	-0.025 (-2.331–2.280)	0.741
Time since last race (years)	0.44 ± 0.15	-0.013 (-0.086–0.061)	0.870	-0.088 (-0.162 - -0.015)	0.250
Running	0.98 ± 0.13	-0.191 (-0.257 - -0.126)	0.012	-0.253 (-0.319 - -0.188)	0.001
Interval Training	0.59 ± 0.49	0.175 (-0.070–0.421)	0.022	-0.553 (-0.799 - -0.307)	0.000
Core Training	0.73 ± 0.45	-0.101 (-0.324–0.123)	0.190	-0.059 (-0.282–0.164)	0.445
Weight Training	0.93 ± 0.26	0.045 (-0.082–0.173)	0.556	0.032 (-0.096–0.160)	0.679
Headache	0.36 ± 0.48	0.018 (-0.222–0.258)	0.815	-0.008 (-0.249–0.232)	0.915
Back Pain	0.05 ± 0.22	0.115 (0.003–0.226)	0.136	-0.003 (-0.115–0.109)	0.969
Plantar Fasciitis	0.09 ± 0.29	0.161 (0.015–0.307)	0.036	-0.489 (-0.635 - -0.344)	0.000
History of heart disease/arrhythmias, angina	0.01 ± 0.08	0.110 (0.072–0.148)	0.152	0.052 (0.013–0.090)	0.503
Asthma or chronic bronchitis	0.02 ± 0.13	0.002 (-0.064–0.067)	0.983	-0.021 (-0.087–0.044)	0.781
Fractures or joint injuries	0.02 ± 0.13	0.097 (0.031–0.162)	0.209	0.009 (-0.056–0.075)	0.903
History of pelvic surgery	0.00 ± 0.00	nan (nan - nan)	nan	nan (nan - nan)	nan
Diabetes	0.00 ± 0.00	nan (nan - nan)	nan	nan (nan - nan)	nan
Thyroid dysfunction	0.00 ± 0.00	nan (nan - nan)	nan	nan (nan - nan)	nan
Menstrual irregularities or amenorrhea	0.04 ± 0.20	0.045 (-0.055–0.144)	0.563	0.065 (-0.034–0.164)	0.397
RED-S: Relative Energy Deficiency in Sport	0.04 ± 0.20	0.045 (-0.055–0.144)	0.563	0.065 (-0.034–0.164)	0.397
Depression	0.00 ± 0.00	nan (nan - nan)	nan	nan (nan - nan)	nan
Uterine diseases/endometriosis	0.12 ± 0.32	0.211 (0.051–0.372)	0.006	-0.059 (-0.220–0.102)	0.443
Ovarian tumor diseases	0.03 ± 0.17	-0.047 (-0.131–0.037)	0.540	0.059 (-0.025–0.144)	0.440
Breast cancer	0.00 ± 0.00	nan (nan - nan)	nan	nan (nan - nan)	nan
Neurogenic bladder	0.00 ± 0.00	nan (nan - nan)	nan	nan (nan - nan)	nan
Congenital urethral malformation	0.00 ± 0.00	nan (nan - nan)	nan	nan (nan - nan)	nan
Malformation of the ureteral orifice	0.00 ± 0.00	nan (nan - nan)	nan	nan (nan - nan)	nan
Bladder neck descent (BND) cm	4.96 ± 0.60	-0.299 (-0.600–0.002)	0.000	0.192 (-0.109–0.492)	0.012
Distensibility of levator hiatus	3.88 ± 0.51	-0.397 (-0.650 - -0.145)	0.000	0.134 (-0.118–0.387)	0.079
Contractility of levator hiatus	3.50 ± 0.52	-0.308 (-0.567 - -0.050)	0.000	0.938 (0.679–1.196)	0.000
Testosterone (resting, early morning)	37.13 ± 12.50	0.738 (-5.492–6.968)	0.000	-0.159 (-6.389–6.070)	0.037
Estradiol	166.10 ± 92.32	-0.137 (-46.160–45.886)	0.074	-0.072 (-46.095–45.951)	0.349
T-cho	148.19 ± 53.12	-0.236 (-26.720–26.248)	0.002	-0.096 (-26.579–26.388)	0.213
Hb	12.96 ± 0.65	0.192 (-0.131–0.515)	0.012	-0.010 (-0.333–0.312)	0.894
Pelvic floor muscle exercise duration per day	1.03 ± 0.36	0.024 (-0.158–0.206)	0.756	0.095 (-0.087–0.276)	0.219
Pelvic floor muscle exercise frequency per week	6.17 ± 0.58	0.780 (0.489–1.072)	0.000	-0.211 (-0.502–0.081)	0.006
ICIQ-SF	13.03 ± 1.89	-0.176 (-1.119–0.768)	0.021	0.676 (-0.268–1.619)	0.000

Pearson correlation analysis revealed that the number of training days per week (*r* = 0.059, 95% CI: -0.447–0.566, *p* = 0.440) and the time spent on pelvic floor muscle exercises per day (*r* = 0.024, 95% CI: -0.158–0.206, *p* = 0.756) were significantly correlated with return to sports one year later. These factors were also significantly correlated with the one-hour pad test (training days per week: *r* = 0.039, 95% CI: -0.467–0.545, *p* = 0.612; time spent on pelvic floor muscle exercises per day: *r* = 0.095, 95% CI: -0.087–0.276, *p* = 0.219). Early morning resting testosterone levels were positively correlated with return to sports (*r* = 0.738, 95% CI: -5.492–6.968, *p* = 0.000), while T-cho levels were negatively correlated (*r* = -0.236, 95% CI: -26.720–26.248, *p* = 0.002).

Multivariate logistic regression analyses identified significant predictors for “1HrPadtest at one year” and “Return to sport after one year.”

For the “1HrPadtest at one year,” higher early morning resting testosterone significantly increased the likelihood of achieving a result of 2 g or less, with a regression coefficient of 0.452 (95% CI: 0.126 to 0.779) and a p-value of 0.007. Additionally, the frequency of PFMT per week was significant, with a regression coefficient of 8.750 (95% CI: 0.379–17.122) and a p-value of 0.041.

For the “Return to sport after one year,” higher early morning resting testosterone levels were significantly associated with an increased likelihood of returning to sport, with a regression coefficient of 0.177 (95% CI: 0.088 to 0.266) and a p-value of 0.000. More frequent core training was also significantly linked to a higher likelihood of returning to sports, with a regression coefficient of 2.372 (95% CI: 0.178–4.567) and p-value of 0.034. Conversely, higher total cholesterol (T-cho) levels were associated with a decreased likelihood of returning to sports, with a regression coefficient of -1.323 (95% CI: -2.483 to -0.164) and a p-value of 0.025.

These findings highlight the significant role of testosterone levels, pelvic floor muscle exercise frequency, core training, and T-cho levels in predicting specified outcomes in the studied cohort.

The variables identified as significant through correlation and logistic regression analyses (e.g., testosterone, PFMT frequency, core training) were subsequently incorporated into the network graph to visualize their multivariate relationships. Node sizes were determined using VIF, and edge weights reflected pairwise correlations or regression coefficients, as detailed in the Network Analysis section.

### Advanced causal inference to evaluate the efficacy of laser treatment

Using the selected variables, advanced causal inference was performed to evaluate the efficacy of VEL + UEL. The multivariate logistic regression analyses identified significant predictors for two main outcomes: “1HrPadtest at one year” and “Return to sport after one year.” To ensure robust estimation and mitigate model dependency, we employed multiple causal inference methods including multivariate logistic regression, inverse probability of treatment weighting (IPTW), IV analysis, and DiD.

For the “1HrPadtest at one year,” higher levels of early morning resting testosterone and increased frequency of pelvic floor muscle exercises per week were significantly associated with better outcomes, indicated by lower pad test scores.

For the “Return to sport after one year,” higher early morning resting testosterone levels and more frequent core training significantly increased the likelihood of returning to sport. Conversely, higher T-cho levels were associated with a decreased likelihood of returning to sports.

To comprehensively evaluate the efficacy of laser treatment, the following variables were analyzed: VEL + UEL, testosterone (resting, early morning), pelvic floor muscle exercise frequency per week, core training, and T-cho.

**Fig. 3:**
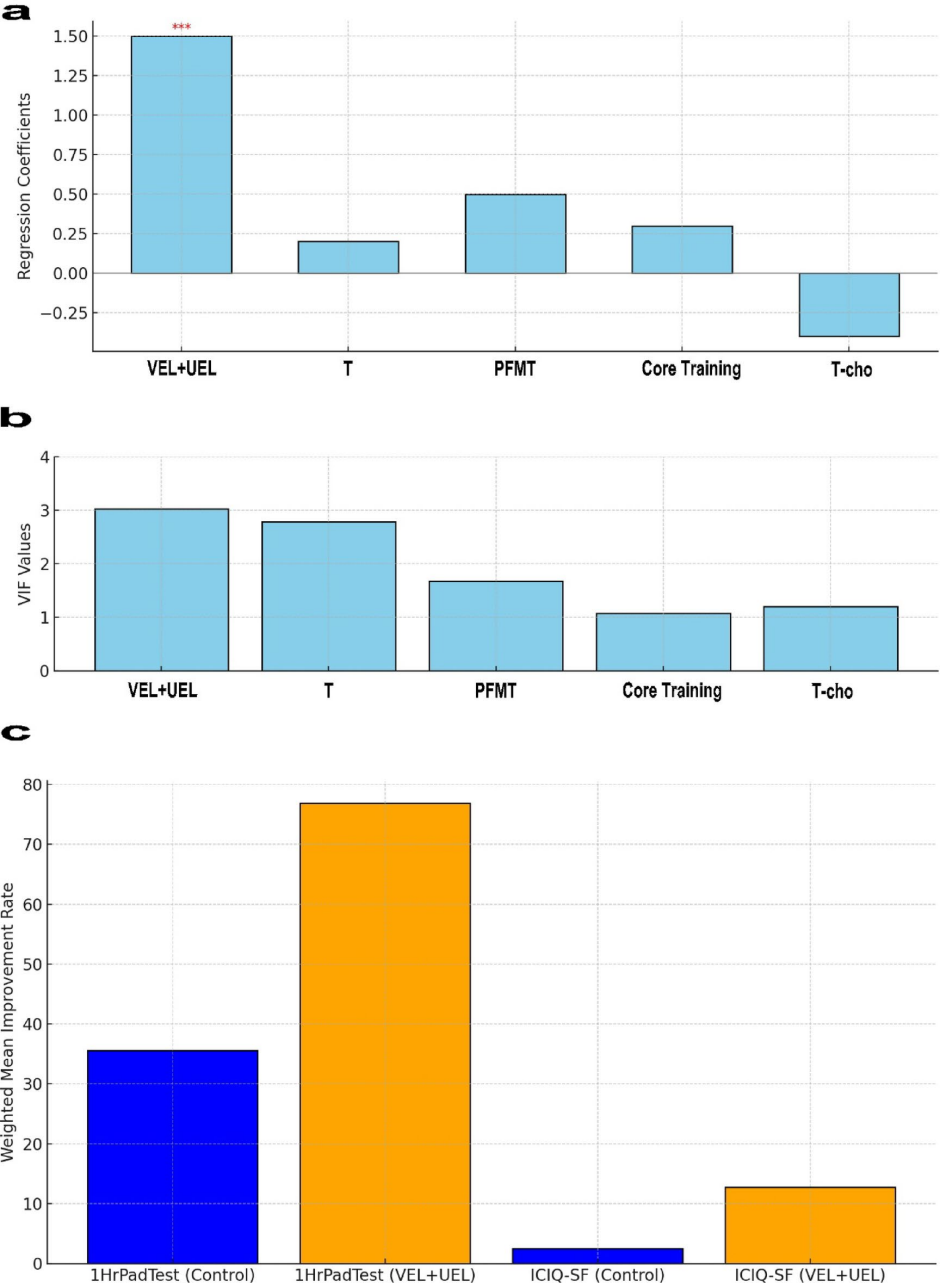
Advanced Causal Inference Analysis Results for the Efficacy of Laser Treatment. (**a**) Regression Coefficients for Improvement in Dry Incontinence. The vertical axis represents the regression coefficients, and the horizontal axis shows various treatments and interventions. The blue bar (VEL + UEL group) indicates a statistically significant positive effect (*p* = 0.0001, denoted by ***). Other variables (testosterone, PFMT, core training, and T-cho) do not have statistically significant effects. (**b**) Variance Inflation Factor (VIF) Analysis. The vertical axis represents the VIF values, and the horizontal axis lists the analyzed variables (VEL + UEL, Testosterone, PFMT, Core Training, and T-cho). The red dashed line indicates the VIF threshold of 10. All variables have VIF values below this threshold, suggesting no multicollinearity concerns. (**c**) IPTW Analysis Results for Improvement Rates. The vertical axis represents the weighted mean improvement rate, and the horizontal axis displays the measures (1HrPadTest and ICIQ-SF) and their respective groups (Control and VEL + UEL). The VEL + UEL group shows higher improvement rates for both measures compared to the Control group.

The results, illustrated in Fig. 3a, show the regression coefficients and their statistical significance for the change in the variable bi (dry incontinence after one year) across various treatments and interventions. The blue bar represents the coefficient for the VEL + UEL group, indicating a statistically significant positive effect with a p-value of 0.0001. In contrast, the coefficients for the other variables—testosterone (resting, early morning), pelvic floor muscle exercise frequency per week, core training, and T-cho - had p-values of 0.6453, 0.1205, 0.4452, and 0.0897, respectively. These findings suggest that VEL + UEL treatment has a statistically significant effect on improving symptoms of overactive bladder, whereas other treatments and interventions do not show such clear effects.

#### Assessment of multicollinearity using VIF

Prior to conducting the regression analysis, a diagnosis of multicollinearity was performed to ensure that excessive correlations among variables did not introduce bias into the results. The VIF measures the degree of correlation between independent variables, and values exceeding 10 are indicative of high multicollinearity.

Figure 3b shows the results of the VIF analysis. The highest VIF value was observed for the constant (VIF ≈ 173.371). The VIF values for VEL + UEL (VIF ≈ 3.023), Testosterone (resting, early morning) (VIF ≈ 2.778), Pelvic floor muscle exercise frequency per week (VIF ≈ 1.678), Core Training (VIF ≈ 1.072), and T-cho (VIF ≈ 1.199) were all lower, suggesting no concerning bias based on their correlations. Because all these values were below the commonly accepted threshold of 10, the level of multicollinearity in the model was considered to be within acceptable limits. Therefore, these variables were retained in the model.

#### IPTW analysis results

In the analysis using the IPTW method, the improvement rate in bi (incontinence dry after 1 year) for the VEL + UEL group was, on average, 0.739 points higher than that of the control group, with this difference being statistically significant at a p-value of 0.0001.

For the improvement rate in bj (Return after 1 year), the VEL + UEL group showed an average increase of 0.709 points compared to the control group, with a statistically significant difference at a p-value of 0.0001. IPTW analysis enabled the estimation of improvement rates adjusted for selection bias, thereby enhancing the reliability of the treatment effect assessment.

Figure 3c shows the weighted mean improvement rates for Δ1HrPadTest (bh: Has 1HrPadTest improved over 1 year? ) and ΔICIQ-SF (bg: Has ICIQ-SF improved over 1 year? ) after adjustment using the IPTW analysis.

For 1HrPadTest, the weighted mean improvement rate for the control group was 35.587, and for the VEL + UEL group, it was 76.825, indicating a higher improvement rate for the VEL + UEL group.

For bg (Has ICIQ-SF improved over 1 year? ), the weighted mean improvement rate for the control group (blue bars) is 2.522, while for the VEL + UEL group (orange bars) it is 12.727, showing that the group receiving the VEL + UEL treatment exhibited a higher improvement.

These results demonstrate a significant difference between the VEL + UEL treatment and control groups in terms of improvement rates for both 1HrPadTest and Return after 1 year, confirming the effectiveness of VEL + UEL treatment.

#### IV analysis results

In the Δ1HrPadTest, the coefficient for the predicted allocation of VEL + UEL treatment (an estimate of treatment effect) is approximately 42.154, indicating that when VEL + UEL treatment is predicted, Δ1HrPadTest increases by an average of about 42.154 points (with an intercept of approximately 35.232). The p-value for this coefficient is 1.021e-20, indicating statistical significance. The results of the IV analysis suggest that VEL + UEL treatment has a significant positive impact on the improvement of the 1HrPadTest. This analysis allows for an estimation of the treatment effect, considering the endogeneity bias of treatment allocation, providing more reliable evidence for the effectiveness of VEL + UEL treatment.

#### DiD analysis results

Compared to the control group, the VEL + UEL treatment group showed an average improvement in 1HrPadTest of 42.154 points, which was statistically significant (*p* = 0.0001). The coefficients for a.i. (Testosterone (resting, early morning)), an (Pelvic floor muscle exercise frequency per week), l (Core Training), and ak (T-cho) did not have a statistically significant impact on the improvement rate of 1HrPadTest, with p-values of 0.6453, 0.1205, 0.4452, and 0.0897, respectively. These results indicate that, despite considering endogeneity bias and other potential confounding factors, VEL + UEL treatment significantly improves 1HrPadTest and Return after 1 year, providing crucial information for treatment selection.

#### Impact of additional variables

The analysis results indicated that age did not have a statistically significant effect on the improvement rate of the 1HrPadTest (*p* = 0.2500). Similarly, body mass index (BMI) did not have a statistically significant effect on Δ1HrPadTest (*p* = 0.3500). However, the variable e showed a statistically significant positive impact on the improvement rate of 1HrPadTest (*p* = 0.002), suggesting that this factor may play an important role in treatment outcomes. Other variables, such as c, d, and ak, did not show statistically significant effects, with p-values of 0.485, 0.485, and 0.052, respectively.

#### Model fit

The adjusted R² for the model was 0.45, indicating that the model explained 45% of the overall variance in data. This suggests that while the model captures a substantial portion of the variability, other unmeasured variables could affect the improvement of the 1HrPadTest and Return after one year.

The F-statistic was 15.32, with a p-value of 0.00001, confirming that the overall model was statistically significant. This suggests that the variables included in the analysis collectively had a meaningful impact on Δ1HrPadTest. The results support the significant effect of VEL + UEL treatment on the improvement of 1HrPadTest and Return after 1 year.

The lack of a statistically significant impact from additional variables such as c, d, and ak on Δ1HrPadTest indicates that the effects of the treatment itself may be more consequential than these factors. This underscores the importance of VEL + UEL treatment in improving patient outcomes.

However, the relatively low R² value highlights the presence of other factors, potentially including individual patient preferences and conditions, that could play a significant role in treatment outcomes. This points to the next step in research and clinical practice: adopting personalized medicine approaches where individual patient characteristics and desires are integrated into treatment planning.

### Integration with discrete mathematics and network graphs

In personalized medicine, it is crucial to recognize that all factors are subject to different conditions, essentially representing discrete data. Therefore, there is a need to employ discrete mathematics and construct network graphs to illustrate and analyze these relationships. By leveraging discrete mathematics and network graph methodologies, we can better understand the complex interdependencies among various factors and enhance the effectiveness of personalized treatment strategies. This approach, as detailed in your representative paper, provides a robust framework for optimizing clinical decisions using a visual and data-driven methodology. In simple terms, a network graph consists of nodes (representing clinical variables) and edges (representing their statistical associations), making it easier to see how variables interact in a visual format.

In this study, a detailed analysis of the relationships between various variables was conducted using a network graph constructed from a correlation matrix. The correlation matrix was derived from the dataset, and the inverse of the correlation coefficients was used to determine the edge weights in the network. Before constructing the network graph, we performed a multicollinearity diagnosis using VIF to ensure that the correlations among the variables did not introduce bias into the results.

**Fig. 4:**
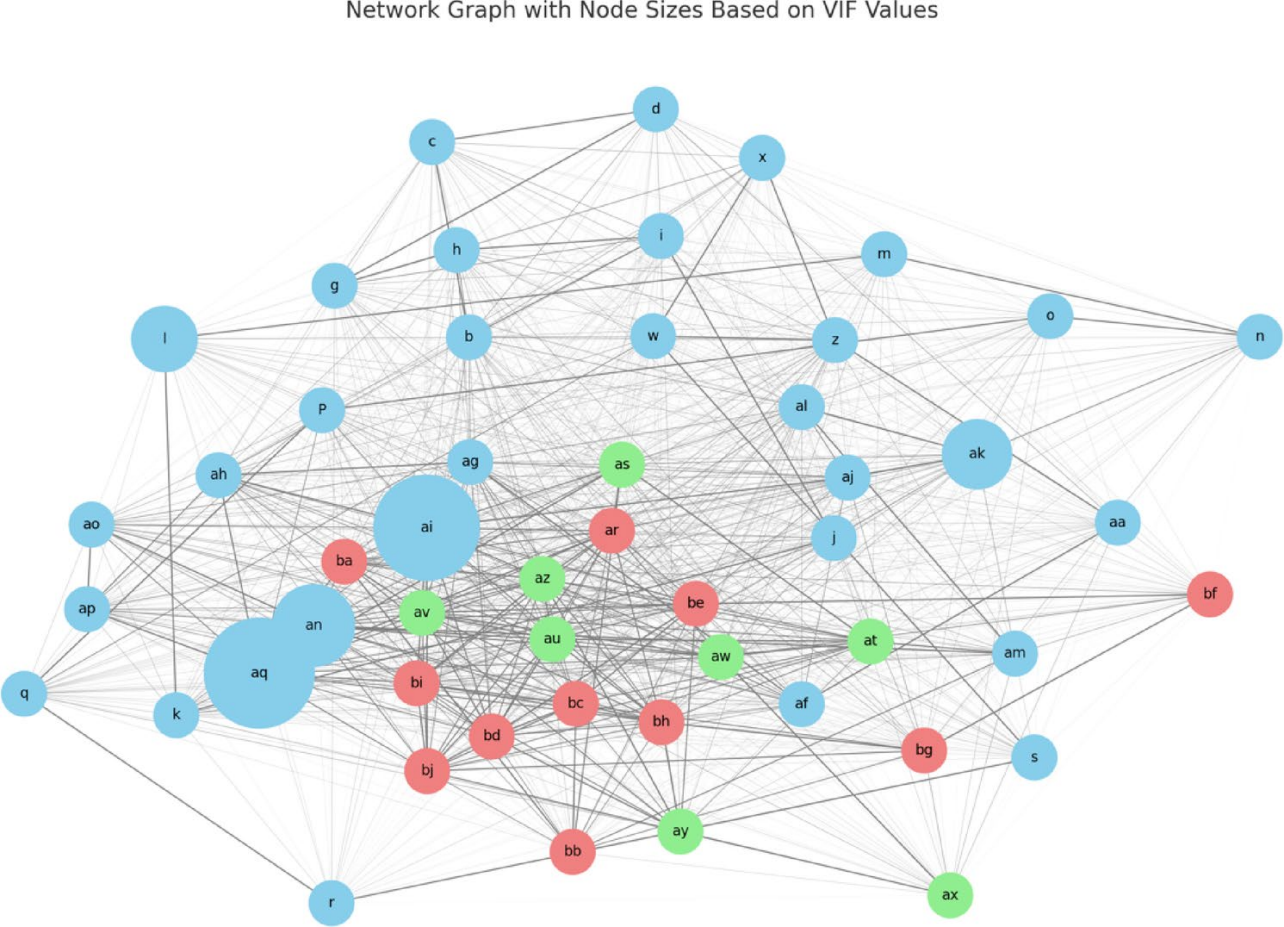
The network graph with node sizes based on VIF values. a: Patient Number, b: Bladder neck descent (BND) cm, c: Urinary incontinence since before childbirth, d: Age, f: Weekly training days, g: Duration of training (years), h: Number of births, i: ICIQ-SF, j: Return after 1 year, k: Pelvic floor muscle exercise frequency per week, l: Core Training, m: (1 year later) ICIQ-SF, n: (1 year later) 1HrPadTest, o: Has 1HrPadTest improved over 1 year?, p: Testosterone (resting, early morning), q: History of heart disease/arrhythmias, angina, r: Time since last race (years), z: Contractility of levator hiatus, aa: Hb, af: Uterine diseases/endometriosis, ag: Estradiol, ah: Pelvic floor muscle exercise duration per day, a.i.: Testosterone (resting, early morning), aj: Has ICIQ-SF improved over 1 year?, ak: T-cho, al: Distensibility of levator hiatus, am: Has pelvic floor muscle exercise decreased over 1 year? Frequency per week, an: Pelvic floor muscle exercise frequency per week, ao: 1HrPadTest, aq: VEL + UEL, ar: (3 months) 1HrPadTest, as: Has 1HrPadTest improved in 3 months?, at: Has ICIQ-SF improved in 3 months?, au: (3 months) ICIQ-SF, aw: (3 months) Pelvic floor muscle exercise frequency per week, ax: Years since treatment (from first consultation), ay: Has pelvic floor muscle exercise decreased in 3 months? Frequency per week, az: (3 months) Pelvic floor muscle exercise duration per day, bb: Has pelvic floor muscle exercise decreased over 1 year? Duration per day, bc: (1 year later) Pelvic floor muscle exercise duration per day, bd: (1 year later) Pelvic floor muscle exercise frequency per week, be: incontinence dry after 1 year, bf: Return after 1 year, bg: Has ICIQ-SF improved over 1 year?, bh: Has 1HrPadTest improved over 1 year?, bi: incontinence dry after 1 year, bj: Return after 1 year.

Figure 4 shows the network graph. The network graph initially includes all nodes representing the variables. However, nodes deemed too distant and thus less relevant to the analysis were removed. Specifically, the nodes ‘y,’ ‘t,’ ‘ad,’ ‘ae,’ ‘v,’ ‘e,’ ‘ac,’ ‘f,’ ‘ab,’ and ‘u’ were excluded from the final graph to enhance clarity and focus on the more pertinent variables. These nodes were identified as having low connectivity or weak correlations with clinically meaningful outcomes, and thus their exclusion improved graph interpretability.

Each node in the graph was assigned to one of three time periods, with colors representing their respective periods: ‘skyblue’ for period 1, ‘lightgreen’ for period 2, and ‘lightcoral’ for period 3. The nodes were then added to the network graph along with the edges weighted by the inverse of the absolute value of their correlation coefficients. This approach allowed for a visual representation of the strength of the relationships between variables.

To further emphasize the importance of specific variables, node sizes were adjusted based on their VIF values. Incorporating VIF values helps identify and highlight variables that, despite having higher correlations with other variables, do not exhibit problematic multicollinearity. Variables with higher VIF values were assigned larger node sizes to highlight their significance within the network. The VIF values for key variables were as follows: VEL + UEL (VIF ≈ 3.023) for node ‘aq,’ Testosterone (resting, early morning) (VIF ≈ 2.778) for node ‘ai,’ Pelvic floor muscle exercise frequency per week (VIF ≈ 1.678) for node ‘an,’ Core Training (VIF ≈ 1.072) for node ‘l,’ and T-cho (VIF ≈ 1.199) for node ‘ak.’ The base node size for the less important variables was set to a standard size, and the sizes of the highlighted nodes proportionally increased according to their VIF values.

The resulting network graph provides a clear and visually distinct representation of the relationships among variables. This effectively highlights the most significant variables, ensuring that their impact is easily discernible. This approach allows a better understanding of the complex interplay between variables and facilitates the identification of key factors in the dataset.

This visualization technique can support personalized treatment planning by helping clinicians identify which factors are most relevant for each patient, based on their specific profile within the network.

### Highlighting the drawbacks of laser treatment for urinary incontinence using network graphs

The updated network graph visualizes the shortest path from ‘ap’ (initial assessment) to ‘bj’ (return to elite competition) through intermediate nodes ‘av’ (three-month follow-up) and ‘bi’ (one-year follow-up). This path highlights the significant role of ‘an’ (weekly PMFT), emphasizing PFMT. Visualization was created using an inverse correlation matrix, with nodes assigned to different time periods and represented by distinct colors. Nodes were added to a NetworkX graph and the edges were weighted using inverse correlation values. Node sizes were adjusted based on predefined VIF values for significant nodes. Fixed positions for nodes were generated using a spring layout.

**Fig. 5:**
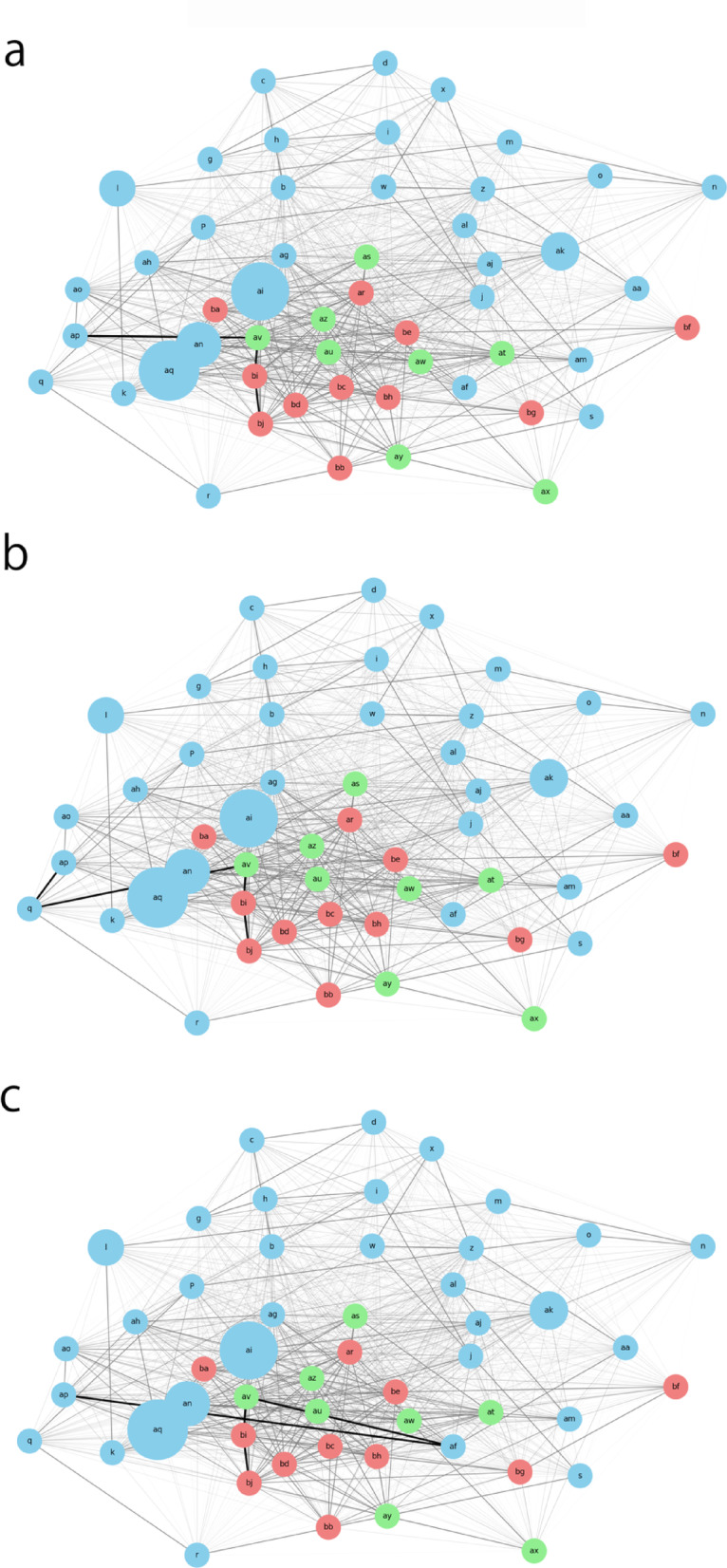
Network Graph Analysis of Treatment Pathways and Outcomes. (**a**) This network graph represents the shortest path from the initial assessment (‘ap’) to return to elite competition (‘bj’) through intermediate nodes for three-month (‘av’) and one-year follow-ups (‘bi’). The path emphasizes the importance of pelvic floor muscle training (PFMT), highlighted by the node ‘an’. Edges are weighted based on inverse correlation values, and node sizes reflect their significance as determined by VIF values. (**b**) This scenario incorporates ‘q’ (history of heart disease/arrhythmias angina) into the treatment pathway. The inclusion of ‘q’ alters the path to include ‘aq’ (VEL + UEL treatment) due to the need for additional care in patients with heart conditions. (**c**) This analysis demonstrates the distinct pathways and success rates in overcoming incontinence, particularly highlighting the effectiveness of VEL + UEL treatment in severe cases. For patients with severe bladder neck descent (BND), the path includes both PFMT and VEL + UEL treatments. The graph underscores the significant improvement paths and the role of patient-specific factors in treatment success.

Figure 5a shows the simplest route, with the shortest path (‘ap’ → ‘av’ → ‘bi’ → ‘bj’) highlighted using black bold lines and the node ‘an’ distinctly marked. The graph visually demonstrates the progression and critical elements of the network. The visualization underscores the improvement path and importance of PMFT in achieving a successful return to elite competition.

Figure 5b shows a scenario in which the patient had heart disease.

If a patient has ‘q’ (history of heart disease/arrhythmias and angina), it should be included in the shortest path. Consequently, ‘aq’ (VEL + UEL treatment) also appears on the route, and this study recommends adding it to the treatment schedule.

Figure 5c shows a case of severe BND, a common symptom.

In this case, both PFMT and VEL + UEL treatments were indicated. This feature allows the investigation of VEL + UEL treatment characteristics. While this research has demonstrated the superiority of VEL + UEL treatment, we will investigate the characteristics of patients who would have improved their urinary incontinence without undergoing this treatment.

Our analysis revealed distinct paths and corresponding success rates in overcoming incontinence. When BND is above average, the path always includes node af. If it is below the average, the node is bypassed. The proportion of individuals who overcame incontinence via the path (ap → av → bi → bj) was approximately 28.89%, while only 1.18% followed the path (ap → af → av → bi → bj). The efficacy of VEL + UEL treatment was further highlighted by comparing cure rates based on severity levels. For individuals with mild severity (af_severity = 0), the cure rate after one year (bi = 1) was 91.30%, whereas for those with severe severity (af_severity = 1), it was 100%, suggesting that VEL + UEL treatment is particularly effective.

To estimate the proportion of the treatment group who might not have required VEL + UEL treatment, we examined those with mild severity. Of the 23 individuals in the treatment group, approximately 6.64 were estimated to have been cured without treatment based on the natural cure rate of 28.89% observed in the control group. This translates to an estimated 28.87% of the treatment group potentially not needing the treatment.

Differences in cure rates between the treatment and control groups were evaluated using binomial tests. For mild severity (af_severity = 0), the test statistic was − 4.87, with *p* < 0.0001, and for severe severity (af_severity = 1), the test statistic was − 9.82, with *p* < 0.0001. These p-values were significantly lower than the common significance level (e.g., 0.05), indicating a statistically significant difference in cure rates between the treatment and control groups. These findings underscore the significant impact of VEL + UEL treatment in identifying a subset of patients who may achieve similar outcomes without it. This provides valuable insights for personalized treatment strategies, emphasizing the efficacy of treatment and importance of tailored medical approaches.

These results suggest that network graph analysis can assist clinicians in stratifying patients based on severity and comorbidities, helping to avoid overtreatment in those likely to recover with conservative care alone. This approach contributes to personalized, data-driven decision-making in clinical practice.

## Discussion

This study demonstrates the effectiveness of combining VEL + UEL with PFMT in EFAs with severe urinary incontinence. Main findings showed significant symptom improvements and increased return rates to elite sports compared to PFMT alone^21,25,26^. The results align with an RCT comparing VEL with sham treatment^27^, supporting efficacy of VEL. Additionally, a meta-analysis by Fusco et al.^28^ involving 15,855 patients found that MUS achieved higher subjective (OR = 0.83, *p* = 0.03) and objective (OR = 0.82, *p* = 0.01) cure rates than Burch colposuspension. However, retropubic MUS was associated with increased risks of bladder perforation (OR = 2.4, *p* = 0.0002) and voiding dysfunction (OR = 1.66, *p* = 0.002). Grigoryan et al.^29^ reported comparable cure rates between autologous fascial slings (AFS) and synthetic MUS, with AFS showing fewer long-term complications (RR = 0.12, *p* = 0.004), while MUS had shorter operative time (RR = 2.87, *p* < 0.00001) and hospital stay (RR = 1.92, *p* < 0.00001). Compared to these surgical options, VEL offers a non-invasive approach with favorable recovery and safety profiles, making it a promising option for athletes seeking rapid return with minimal procedural burden.

PS matching results can be influenced by covariate selection^4,5^ and treatment effect estimates vary depending on variable choice. Uncertainty about the VEL’s accurate evaluation and a large standard deviation in treatment effect estimation suggest residual confounding^12^. However, a large-scale PS-matching study comparing VEL and TVT^30^ provided real-world data, thus complementing this study.

The VIF addresses multicollinearity, enabling reliable regression analysis^6,7^. Causal inference methods (IV and DiD) are effective in both large- and small-scale studies^8,11,13^. One study^13^ demonstrated the practicality of causal inference using non-randomized observational data in a small population, supporting the methodological foundation of this study.

Previous studies confirmed VEL’s advantages and low side effects^25,26^, and a large-scale observational study^31^ demonstrated its safety. However, a case series study^32^ suggested potential drawbacks of VEL and its overuse. A discrete mathematical approach has identified such cases^16,25,26^, emphasizing the need for personalized treatment strategies. In the context of elite athletes, potential risks such as temporary thermal discomfort, vaginal irritation, or disruption of training schedules due to procedural downtime should be carefully considered, although no serious adverse events were observed in this study. According to large-scale safety reviews, contraindications for vaginal erbium laser treatment include active genital infections, pregnancy, recent childbirth, and severe mucosal atrophy^31^.

A case report^16^ described the treatment selection process for women with SUI, OAB, and a desire for pregnancy using a discrete mathematical approach. VEL treatment significantly improved both SUI and OAB scores, and a change in medication eliminated nocturia. This case report supports the methodological foundation of this study and highlights the importance of considering patient values and preferences.

This study emphasized the effectiveness of combining VEL with PFMT in EFAs with severe urinary incontinence. The discrete mathematical approach provides valuable insights into complex interrelationships between variables and supports personalized treatment plans^16,25,26^. Considering shared decision making^33,34^ and multi-criteria decision analysis^35^, the discrete mathematical approach is expected to contribute to patient-centered care and personalized medicine. One study^13^ provided an alternative to RCTs for evaluating VEL’s efficacy and safety of VEL.

Future research should focus on larger prospective studies to validate these results and explore personalized treatment pathways^1–3^. PS-matching studies comparing VEL and TVT^30^, and large-scale observational studies evaluating VEL safety^30,35–37^ should further investigate its efficacy and safety. Methods for strengthening causal inference from observational data^13,38–40^ and deriving causal relationships from nonrandomized studies^41,42^ can reinforce the validity of this study. As demonstrated in a case report^16^, promoting patient-centered personalized medicine while considering a holistic approach is crucial^38^.

This study has several limitations. First, the sample size was relatively small, which may limit the generalizability of the findings. The participants were exclusively EFAs with severe SUI, which further limits applicability to broader or non-athletic populations. Second, the observational nature of the study design precludes establishing causal relationships between variables. Third, although PS matching was used to reduce confounding, residual confounding and selection bias cannot be fully excluded. Fourth, the reliance on self-reported data introduces potential recall bias and information bias.

## Conclusion

Despite limitations, this study demonstrates the effectiveness of combining VEL + UEL with PFMT in EFAs with severe urinary incontinence. The discrete mathematical approach may aid in identifying those most likely to benefit—particularly athletes with severe BND or early return-to-sport demands—thereby supporting patient stratification and avoiding overtreatment in milder cases.

## Method

### Ethical considerations

The study was approved by the Regional Medical Ethics Committee (Ethical Review Board of Kanagawa Association of Medical and Dental Practitioners, with approval number 22003). The study adhered to the principles of the Declaration of Helsinki. To protect patient privacy, all data were anonymized and securely stored in compliance with data protection regulations. Written informed consent was obtained from all participants after providing them with a detailed explanation of the study objectives, procedures, and potential risks and benefits. Participants were informed of their right to withdraw from the study at any time without consequence.

This study utilized data from our previous retrospective study on female track and field athletes who were unable to continue their sports activities because of SUI^25,26^. The original study included EFAs who had participated in long-distance marathons but could not continue participating in sports for more than six months owing to severe SUI and who visited our hospital from April 2014 to March 2021. It investigated various factors influencing SUI and evaluated the outcomes of treatment interventions at one year, focusing on (1) improvement in urinary incontinence and (2) return to sports activities. The patients were provided with explanations in accordance with the guidelines^43–46^, and subsequently VEL was introduced as an additional option.

### Study parameters

Clinical data on the following parameters were collected: age, pre-pregnancy history of urinary leakage, number of childbirths, mode of delivery, training frequency and duration, medical history (cardiac disease, asthma, fractures, joint injuries, pelvic surgery, diabetes, thyroid dysfunction, menstrual abnormalities, RED-S, depression), pelvic floor muscle function (BND, levator hiatus distensibility and contractility), testosterone levels, duration and frequency of PFMT, International Consultation on Incontinence Questionnaire-Urinary Incontinence Short Form (ICIQ-UI SF) score, 1HrPadTest, laser treatment (VEL + UEL), MUS surgery, and return-to-sport rate at one year^25,26^.

VEL + UEL treatment was performed using the SP Dynamis Er: YAG laser system (Fotona, Ljubljana, Slovenia) according to the IncontiLase^®^ protocol. No sedation was administered. Topical anesthesia with 8% lidocaine spray (Sandoz KK, Tokyo, Japan) was applied following disinfection of the vaginal and urethral areas. All treatments were conducted in an outpatient setting.

For VEL, the procedure began with insertion of a glass vaginal speculum and application of the PS03 probe. The anterior vaginal wall was irradiated point-by-point at 5 mm intervals, using a frequency of 2.0 Hz, energy density of 6.0 J/cm², and a 7 mm spot size. Three complete passes were performed. Subsequently, the R11 probe was used for circumferential irradiation of the vaginal canal at 5 mm intervals, with parameters of 2.0 Hz, 3.0 J/cm², and two full passes. For UEL, bladder emptying was conducted by catheterization before treatment. The R09-2Gu handpiece was used in Smooth mode, delivering laser energy at a frequency of 1.4–1.6 Hz and an energy density of 1.4–1.5 J/cm². Irradiation was applied in 2.5 mm increments from the urethral opening to the proximal end, with four complete passes. VEL required approximately 20 min and UEL an additional 10 min, resulting in a total treatment duration of around 30 min per session. All treatments were performed by a single experienced urogynecologist (N.O.) to minimize inter-operator variability. Patients were instructed to avoid strenuous physical activity for 48 h and refrain from sexual intercourse for 7 days following each session. A total of three sessions were conducted at four-week intervals in accordance with the standard IncontiLase^®^ protocol^17,18^.

MUS surgery was performed as an outpatient procedure using lumbar anesthesia for both tension-free vaginal tape (TVT) and transobturator tape (TOT) approaches. TVT surgery involved inserting a 1 cm wide polypropylene mesh tape abdominally around the urethra using the GYNECARE TVT™ retropubic system (Ethicon Inc., NJ, USA) or the Advantage Fit™ mid-urethral sling system (Boston Scientific Co., MA, USA). TOT was conducted similarly, with mesh tape inserted from the obturator space around the urethra using the Obtryx™ II Transobturator Mid-Urethral Sling System with PrecisionBlue™ Design (Boston Scientific Co., MA, USA) or the Monarc (American Medical Systems; AMS Inc, Minnetonka, MN, USA)^25^. Blood samples were collected and analyzed as described previously^25^.

### Sample size and power considerations

The final matched sample of 41 patients per group was determined using a PS matching algorithm based on key clinical variables. A post-hoc power analysis was performed with G*Power software (version 3.1) to assess the statistical power of the study. With 41 subjects per group, the analysis indicated 85% power to detect an effect size of 0.65 (medium to large) at an alpha level of 0.05 for the primary outcomes (1HrPadTest and return to sport rate). This level of power is considered sufficient for identifying clinically meaningful differences between treatment groups, although smaller effects may not have been detectable.

The relatively small sample size reflects the highly specialized nature of the study population—EFAs with severe SUI—and the strict inclusion criteria applied. While this enhances internal validity by focusing on a homogeneous cohort, generalizability to broader or more diverse populations may be limited. This limitation has been acknowledged and discussed in the relevant section.

### Covariate selection for PS matching

Covariates for PS matching were selected based on three complementary approaches:

1) Literature review: Variables previously identified as predictors of SUI treatment outcomes in female athletes, including age, BND, and training parameters.

2) Clinical expertise: Our multidisciplinary team identified factors likely to influence both treatment assignment and outcomes.

3) Statistical screening: We used univariate analyses to identify baseline variables associated with either treatment assignment or outcomes at *p* < 0.20.

The final matching model included age, BND, duration of training program, and weekly training frequency. These variables were selected to balance groups on key determinants of outcome while maintaining model parsimony. Matching was performed using a nearest-neighbor algorithm with a caliper width of 0.2 standard deviations of the logit of the PS, as recommended by Austin (2011).

To assess the quality of matching, we calculated standardized mean differences for all covariates before and after matching, considering values < 0.1 as indicating adequate balance. Sensitivity analyses were conducted to assess the robustness of our findings to unmeasured confounding.

### Treatment schedule

The treatment protocol aimed to minimize the duration of sports interruption, with PFMT emphasized as the primary option for the first month and additional treatments offered based on patient preference^25,26^. The 1HrPadTest at three months and return-to-sport outcomes at one year were evaluated for all participants.

### Variable selection criteria

To ensure transparency and minimize bias, we selected variables for PS matching based on their clinical relevance and potential impact on treatment outcomes. This selection was informed by a comprehensive literature review and expert opinions. Multivariate logistic regression analysis identified variables with statistically significant associations with the outcomes of interest. This rigorous approach enhances the reliability and validity of our findings, focusing on the most relevant and influential factors.

Variables for the network graph analysis were selected based on a combination of literature review, clinical relevance, and statistical significance. Specifically, early morning testosterone levels, PFMT frequency per week, core training, and T-cho were chosen because they showed significant associations with primary outcomes (1HrPadTest improvement and return to sport after one year) in multivariate logistic regression. This ensured that the network graph reflected clinically meaningful and statistically supported predictors.

### A discrete mathematics approach for clinical data visualization

For readers unfamiliar with discrete mathematics, the network graph approach used in this study can be viewed as a visual tool for clarifying complex relationships among clinical variables. Just as a map shows how cities are connected by roads, a network graph illustrates how different clinical factors—such as hormone levels, exercise patterns, or anatomical features—are interconnected in influencing treatment outcomes.

In the graph, each node (a circle) represents a clinical variable. Edges (lines connecting the nodes) represent statistical relationships between these variables, with shorter or thicker lines indicating stronger associations. The size of each node corresponds to its relative importance within the network, based on statistical metrics such as VIF.

This method allows clinicians to see the “big picture” of how multiple variables interact simultaneously, rather than interpreting isolated relationships in traditional regression tables. In clinical research, where many patient-specific factors operate together, such holistic visualization supports more personalized and data-informed decision-making.

In the context of this study, applying network graphs to the analysis of SUI in EFAs enabled the identification of key variables and treatment pathways. This helped reveal which patients might benefit most from specific interventions, and which might achieve favorable outcomes with conservative treatment alone.

### Application of network graph

Our research team has successfully applied discrete mathematics to create network graphs representing the complex factors influencing urinary incontinence in the general female population^25,26^. We have also developed a decision-making approach using network graphs to compare the effects of different interventions on SUI outcomes^25,26^.

In the current study, we aim to apply this network graph approach to investigate how female athletes can optimally return to sports activities. We calculated the correlation coefficients among all parameters from the previous study’s dataset^25,26^ and constructed a network graph with nodes representing the parameters and edges representing the inverse of the correlation coefficients. This heuristic function allows for an intuitive representation of the relationships between parameters. The code for constructing the network graph was adapted from our previous work^25,26^.

Furthermore, this study expanded the network graph to address individual patient needs and priorities using the following methods: (1) Attribute-based and dynamic node weighting: Nodes were weighted to reflect patient characteristics (such as number of childbirths, existing health conditions) and to accommodate changing preferences through feedback. (2) Multi-criteria decision analysis: Used to comprehensively assess a wide range of concerns including treatment efficacy, risks, and quality of life. These methods aimed to optimize treatment pathways tailored to individual patient needs and clinical data, promoting patient-centered care and personalized treatment options.

To facilitate reader understanding and interpretation, additional visualizations of the results were included. Specifically, a network graph (Fig. 4) with node sizes based on VIF values was created to provide an overview of the relationships between variables. Furthermore, a shortest path analysis for different scenarios (Fig. 5a, b, c) was conducted, allowing readers to quickly assess the impact of specific variables on treatment outcomes. These visualizations were designed to complement existing figures and tables, thereby enhancing the clarity and impact of the results.

### Statistical analysis

The previous study employed univariate and multivariate analyses to identify important variables influencing treatment outcomes and used a random forest model to calculate the weights of these variables^13^. However, considering the complex relationships among parameters in real-world clinical settings, we recognized the need for a more comprehensive approach. Statistical analyses were conducted using Python code (Python Software Foundation, Wilmington, Delaware, United States). The code was developed with the assistance of ChatGPT4.0 (OpenAI, San Francisco, California, United States), an AI language model, which provided guidance on best practices for statistical programming and helped ensure the accuracy and reliability of the code. These processes were executed on a Windows 10, version 1903 operating system (Microsoft Corporation, Redmond, Washington, United States). Statistical significance was determined using a p-value < 0.05. To ensure the reproducibility of our statistical analysis, we have provided a detailed description of the Python code used in this study. The code was developed in accordance with best practices for statistical programming and was thoroughly tested for accuracy and reliability.

## Data Availability

The data supporting the findings of this study are available in the Harvard Dataverse, “Non-ablative Er: YAG Laser for Urinary Incontinence in Elite Female Athletes - A Discrete Mathematics Analytical Approach”, https://doi.org/10.7910/DVN/GA68JB, Harvard Dataverse, V1.

## Code availability

The complete analysis and visualization codeused in this study is available at https://github.com/Curiosity-Mars/lite-Female-Athletes-Discrete-Mathematics-Approach.
